# The role of c-Met and VEGFR2 in glioblastoma resistance to bevacizumab

**DOI:** 10.1038/s41598-021-85385-1

**Published:** 2021-03-16

**Authors:** Bruno Carvalho, José Manuel Lopes, Roberto Silva, Joana Peixoto, Dina Leitão, Paula Soares, Ana Catarina Fernandes, Paulo Linhares, Rui Vaz, Jorge Lima

**Affiliations:** 1 Faculty of Medicine, University of Porto, Alameda Prof. Hernâni Monteiro, 4200-319 Porto, Portugal; 2 Department of Neurosurgery, Centro Hospitalar Universitário S. João, Alameda Prof. Hernâni Monteiro, 4200-319 Porto, Portugal; 3 Department of Pathology, Centro Hospitalar Universitário S. João, Alameda Prof. Hernâni Monteiro, 4200-319 Porto, Portugal; 4 Department of Oncology, Centro Hospitalar Universitário S. João, Alameda Prof. Hernâni Monteiro, 4200-319 Porto, Portugal; 5 Instituto de Investigação E Inovação Em Saúde (i3S), Porto, Portugal; 6 Institute of Molecular Pathology and Immunology, University of Porto (Ipatimup), Porto, Portugal; 7 Neurosciences Center-CUF Hospital, Estrada da Circunvalação 14341, 4100-180 Porto, Portugal

**Keywords:** CNS cancer, Tumour angiogenesis, Tumour biomarkers

## Abstract

Dismal prognosis of glioblastoma (GBM) prompts for the identification of response predictors and therapeutic resistance mechanisms of current therapies. The authors investigated the impact of c-Met, HGF, VEGFR2 expression and microvessel density (MVD) in GBM patients submitted to second-line chemotherapy with bevacizumab. Immunohistochemical expression of c-Met, HGF, VEGFR2, and MVD was assessed in tumor specimens of GBM patients treated with bevacizumab, after progression under temozolomide. Survival analysis was evaluated according to the expression of the aforementioned biomarkers. c-Met overexpression was associated with a time-to-progression (TTP) after bevacizumab of 3 months (95% CI, 1.5–4.5) compared with a TTP of 7 months (95% CI, 4.6–9.4) in patients with low or no expression of c-Met (*p* = 0.05). VEGFR2 expression was associated with a TTP after bevacizumab of 3 months (95% CI, 1.8–4.2) compared with a TTP of 7 months (95% CI, 5.7–8.3) in patients with no tumoral expression of VEGFR2 (*p* = 0.009). Concomitant c-Met/VEGFR2 overexpression was associated with worse overall survival (13 months) compared with concomitant c-Met/VEGFR2 negative expression (19 months; *p* = 0.025). Our data support the hypothesis that c-Met and VEGFR2 overexpression have a role in the development of glioblastoma early resistance and might predict poorer responses to anti-angiogenic therapies.

## Introduction

Despite all therapeutic and research efforts, glioblastoma (GBM) prognosis remains ominous with a median overall survival (OS) of 14.6 months and a 5-year survival rate of 9.8% in the temozolomide era^1^. Although median OS has improved to 20.9 months with the recent addition of alternating electric field therapy^2^, progression is inexorable and treatment options for recurrency are scarce, with second-line chemotherapy showing only modest anti-tumoral activity^3^.

Bevacizumab is a recombinant human monoclonal antibody that selectively neutralizes VEGF, which showed improved progression-free survival (PFS) and objective response rates, leading to the US Food and Drug Administration approval for recurrent GBM in 2009^4,5^. Although subsequent antiangiogenic phase III clinical trials did not validate the results concerning OS in newly diagnosed and recurrent GBM^6–8^, bevacizumab remains a cornerstone therapy in the treatment of recurrent GBM, providing symptom alleviation, increased PFS and probably a valid survival benefit, in a subgroup of patients^9–12^.

Defining populations of patients who can benefit most from a particular therapy will improve cost-effectiveness and therapeutic outcomes, avoiding futile or harmful treatments^13^. In this perspective, it is crucial to identify response predictors and therapeutic resistance mechanisms of presently available therapies in GBM patients. There are emerging systemic, circulating, tissue and imaging biological markers, but no validated biomarkers for appropriately selecting GBM patients who might respond to antiangiogenic therapy. While survival benefit from bevacizumab-containing regimens is modest, some patients respond better than others and it is crucial to establish predictive and therapy monitoring markers^14^.

Resistance to antiangiogenic therapies is partly due to a substantial redundancy in proangiogenic ligands and receptors. Evidence suggests that, in the early phase, tumors are dependent on VEGF and resistance emerges from the upregulation of HGF/c-MET pathway^15,16^. In this standpoint, our purpose was to evaluate the prognostic and therapeutic significance of c-Met, HGF and VEGFR2 expression, together with microvessel density (MVD), in patients with recurrent GBM undergoing second-line chemotherapy with bevacizumab-based therapy.

## Methods

### Patient and tissue collection

We conducted a retrospective study of 40 consecutive patients with recurrent GBM treated at Centro Hospitalar Universitário São João, Oporto, between January 2008 and October 2013. All patients were treated with radio-chemotherapy according to the Stupp protocol, followed by treatment with bevacizumab-based therapy, upon recurrence.

The inclusion criteria were (i) patients with histologically proven GBM, age ≥ 18 years; (ii) recurrence after magnetic resonance imaging response assessment; and (iii) second-line treatment with bevacizumab-based regimen after neuro-oncology multidisciplinary team meeting decision.

All patients were treated according to standard first-line chemotherapy with temozolomide, administered 75 mg/m^2^ concurrent with daily external-beam radiation therapy (RT) (2 Gy/fraction, for a total of 60 Gy in 30 fractions) and followed by adjuvant TMZ at 150–200 mg/m^2^ for 5 days every 28 days until progression. All patients were treated with second-line bevacizumab-based therapy, namely bevacizumab (10 mg/kg) + irinotecan (340 or 125 mg/m^2^, with or without concomitant enzyme inducing antiepileptic drugs, respectively) every two weeks.

Two neuropathologists (JML/RS) retrospectively reviewed the diagnoses after collection of paraffin-embedded tumor specimens. Subsequently, the immunohistochemical (IHC) evaluation of c-Met, HGF, VEGFR2, and MVD (CD31 expression) was performed in representative tumor sections. All cases were *IDH*-wild type as assessed by Sanger sequencing (*IDH1* and *IDH2*).

Demographic, clinical, therapeutic and survival data of all patients were collected and analysed. Then, the tissue expression of the aforementioned biomarkers was correlated with survival data, namely OS, time to progression after temozolomide (TTP-1), and time to progression after bevacizumab (TTP-2).

This study was approved by the Local Ethical Committee of Centro Hospitalar de São João (Porto, Portugal) and conducted according to the National Ethical Guidelines.

### Immunohistochemistry study

Serial tissue sections (3 µm thick) of representative tumor samples were prepared on the slide for IHC analysis. Sections were immunostained with the following monoclonal antibodies: c-Met (18-2257, Invitrogen), HGF (H-145:SC-7949 Santa Cruz Biotechnology), VEGFR2 (55B11, Cell Signaling) and CD 31 (JC70 Cell Marque).

For the c-Met IHC, we performed deparaffinization and rehydration of tissue sections, followed by heat-induced antigen retrieval (citrate buffer [pH 6.0] 2 min at 65 ºC, 5 min 125 ºC in a steamer) and blockage of endogenous peroxidase activity (0.3% hydrogen peroxide in methanol). Incubation with the primary antibody against c-Met (dilution 1:100) was conducted overnight at 4 ºC, followed by biotinylated secondary antibody, streptavidin-HRP and staining with diaminobenzidine (DAB) chromogen (Dako Carpinteria, CA, USA) and counterstained with haematoxylin. The positive control was a sample of papillary renal carcinoma.

For the HGF IHC, we performed deparaffinization and rehydration of tissue sections, followed by heat-induced antigen retrieval (EDTA buffer [pH 9.0] 5 min at 98 ºC in a steamer) and blockage of endogenous peroxidase activity (0.3% hydrogen peroxide in methanol). Incubation with the primary antibody against HGF (dilution 1:50) was conducted over-night at 4 ºC, followed by the same procedure mentioned above. The positive control was a colon sample.

VEGFR2 and CD31 immunostaining was performed using the Ventana Benchmark Ultra automated immunostainer (Ventana Medical System, Tucson, AZ, USA). Briefly, the tissue sections were deparaffinized using EZPrep (VMS, USA) at 72 ºC, and washed with Reaction Buffer (VMS, USA). Antigen retrieval was achieved using Cell Conditioning solution 1 (CC1 Universal Buffer Tris/EDTA pH  8.4) (VMS, USA), at a temperature ranging from 95 to 98 ºC, followed by blockage of endogenous peroxidase activity using OptiView Peroxidase Inhibitor (3.0%). Diluent Option (VMS, USA) was also used to decrease unspecific background signal. Primary antibodies were then incubated using the optimized dilutions and time (CD31 antibody 1/100 dilution and 32 min incubation and VEGFR2 antibody 1/600 dilution and 1 h 30 min incubation). The detection of the primary antibody was accomplished using the OptiView Horseradish Peroxidase Universal Multimer (VMS, USA) and the signal revelation was obtained with OptiView cocktail (VMS, USA)-DAB, H2O2 and Copper. Sections were counterstained with Hematoxylin and Bluing Reagent (VMS, USA) and washed in tap water with EZPrep, to remove oil coverslip. Finally, slides were dehydrated using a series of ethanol and xylene and mounted for light microscopy. The specificity of immunohistochemistry was checked using negative and positive controls.

### Scoring and interpretation of immunohistochemistry

Sections were examined for c-Met, HGF, VEGFR2 and CD31 immunoreactivity by two neuropathologists blinded to the outcomes and clinical features. Tumors were classified according to the expression intensity and extension, which were then used to reach a composite score (intensity × extension). For the intensity, tumors were classified as slight or no expressing (grade 1 or 0, respectively), moderate expression (grade 2) or strong expression (grade 3). Regarding extension, tumors were classified according to the percentage of positive cells: staining observed in less than 25% of cells (grade 1), in a sizable subgroup of cells (25–50%; grade 2), most cells (50–75%; grade 3), or nearly all cells (> 75%; grade 4) in the high-power field. Tumors displaying moderate immunopositivity in > 50% of tumor cells or strong immunopositivity in > 25% of tumor cells were considered to be positive tumors; all other cases were considered negative. For statistical purposes, immunoreactivity was analyzed as a dichotomous covariate classified as negative or weak versus positive or strongly positive^17^.

For evaluating MVD, the three most vascularized areas (hot spots) were selected in sections stained for CD31, using low-power field (× 40) magnification. The counting of vessels was performed in these areas, using high-power field (× 200, 0.1 mm^2^) and avoiding areas of sclerosis, necrosis, and non-neoplastic tissue ^18,19^. The number of CD31-positive vessels/0.1 mm^2^ of tumor tissue was calculated in three hotspots and its average was used to indicate MVD.

### Data and statistical analysis

The primary endpoint of this study was to correlate the expression of c-Met, HGF, VEGFR2 and the MVD with time to progression after bevacizumab (TTP-2), and secondarily with time to progression after temozolomide (TTP-1) and OS. Differences between groups were analysed using Chi-square, Fisher’s exact test or Mann–Whitney U test as appropriate. Survival data was evaluated using Kaplan–Meier product-limit analysis and 95% confidence intervals (CI) were calculated. The Log-rank test was used to detect statistically significant differences in survival distribution. Multivariate survival analysis was calculated using the Cox proportional hazard method to settle on independent prognostic factors. Differences were considered statistically significant at P < 0.05. The software used for statistical analysis was IBM SPSS Statistics 25.

### Ethics approval and consent to participate

This study was approved by the Local Ethical Committee of Centro Hospitalar de São João, (Porto, Portugal)/Medical Faculty (Porto, Portugal) and was conducted according to the National Ethical Guidelines. Informed consent was waived by the Local Ethical Committee of Centro Hospitalar S. João/Faculty of Medicine of the University of Porto (Comissão de Ética para a Saúde do Centro Hospitalar S. João/Faculdade de Medicina da Universidade do Porto). The study was performed in accordance with the Declaration of Helsinki.

## Results

### Patients demographics and clinical outcome

The summary of demographic, clinical, therapeutic and survival data of the cohort is presented in Table 1. The median age at diagnosis was 59 years (42–73). The majority of patients were male (70%, n=28) and 90% (n=36) had an ECOG performance status of 0 or 1. Complete resection was achieved in 22 patients (55%), partial resection in 12 patients (30%) and biopsy in 6 patients (15%). Median OS was 18 months (95% CI 16.2-19.8) for the entire cohort. The median time to progression after temozolomide (mTTP-1) was 8 months (95% CI 6.8-9.2) and the median time to progression after bevacizumab (mTTP-2) was 5 months (95% CI 2.9-7.1).

**Table 1:** Clinical and demographic characteristics of GBM patients.

Variables	N (%)
**Gender**	
Male Female	28 (70) 12 (30)
Age (years), median Range	59 42–73
**ECOG**	
0 1 2 3	21 (52.5) 15 (37.5) 2 (5.0) 2 (5.0)
**Focality**	
Unifocal Multifocal	37 (92.5) 3 (7.5)
**Type of surgery**	
Total resection Partial resection Biopsy	22 (55) 12 (30) 6 (15)
Number of TMZ cycles, median Range	5 1–28
mOS, months 95% CI	18 16.2–19.8
mTTP-1, months 95% CI	8 6.8–9.2
mTTP-2, months 95% CI	5 2.9–7.1

*ECOG* Eastern Cooperative Oncology Group performance status, *TMZ* temozolomide, *mOS* median overall survival, *mTTP-1* median time to progression after temozolomide, *mTTP-2* median time to progression after bevacizumab, *95% CI* 95% confidence interval.

### Biomarker expression and survival analysis

The expression of biomarkers was detected as follows: c-Met overexpression was detected in 36.8% (14 of 38) of the patients, HGF overexpression was detected in 35.3% (12 of 34) of the patients and VEGFR2 overexpression was detected in 25.6% (10 of 39) of the patients.

**Figure 1:**
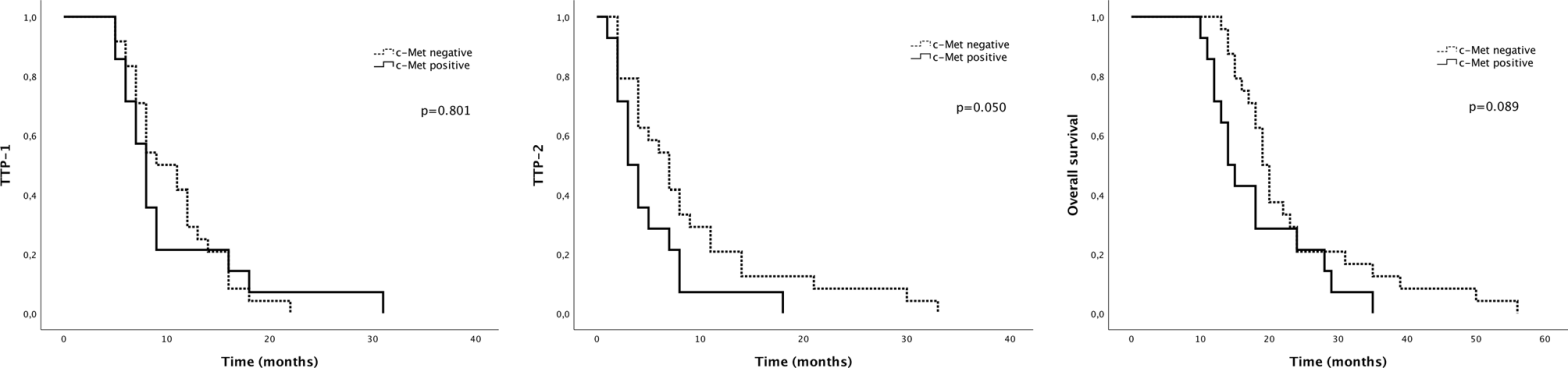
Kaplan–Meier analysis of survival curves according to c-Met expression.

The expression of c-Met had no impact on TTP-1. Patients with GBM overexpressing c-Met had a mTTP-2 of 3 months (95% CI 1.5–4.5) versus 7 months (95% CI 4.6–9.4) in patients with GBM with little or no expression of c-Met expression (Log-rank test, p = 0.050). There was a trend towards better median OS in patients with c-Met negative tumors (19 months, 95% CI 17.4–20.6) versus patients with c-Met positive tumors (14 months, 95% CI 11.6–16.5) (Log-rank test, p = 0.089) (Fig. 1).

**Figure 2:**
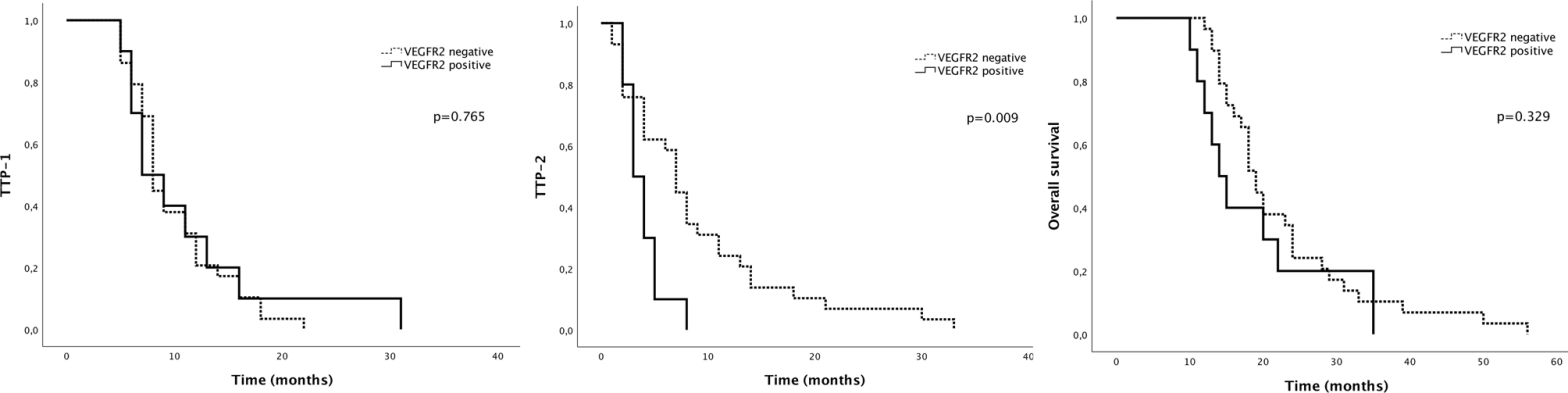
Kaplan–Meier analysis of survival curves according to VEGFR2 expression.

The results for VEGFR2 expression were similar to c-Met: while there was no impact on TTP-1, the TTP-2 of patients with VEGFR2-overexpressing GBM was significantly shorter than the TTP2 of patients with VEGFR2-negative GBM (3 months versus 7 months, respectively, p = 0.009). In VEGFR2-negative group the response rate was 62% (18 out of 29 patients) versus 10% (1 out of 10 patients) in VEGFR2-positive group (Fisher’s exact test, p = 0.008). There was no difference in median OS between patients with VEGFR2-positive and negative tumors (14 months versus 19 months, respectively, p = 0.329) (Fig. 2).

Concerning HGF, there were no significant differences between patients with HGF-positive and negative tumors.

The number of CD31-positive vessels/0.1 mm^2^ of tumor tissue was used to calculate MVD. The median value of MVD was 17.65 (range 2.7–36.3) vessels/ 0.1 mm^2^. We did not find any statistically significant difference between patients with higher (above median) MVD and lower MVD regarding TTP-1, TTP-2 or OS. High MVD group showed TTP-1 of 9 months (versus 8 months in low MVD group, p = 0.535), TTP-2 of 7 months (versus 4 months in low MVD group, p = 0.297) and an OS of 19 months (versus 16 months in low MVD group, p = 0.090).

**Figure 3:**
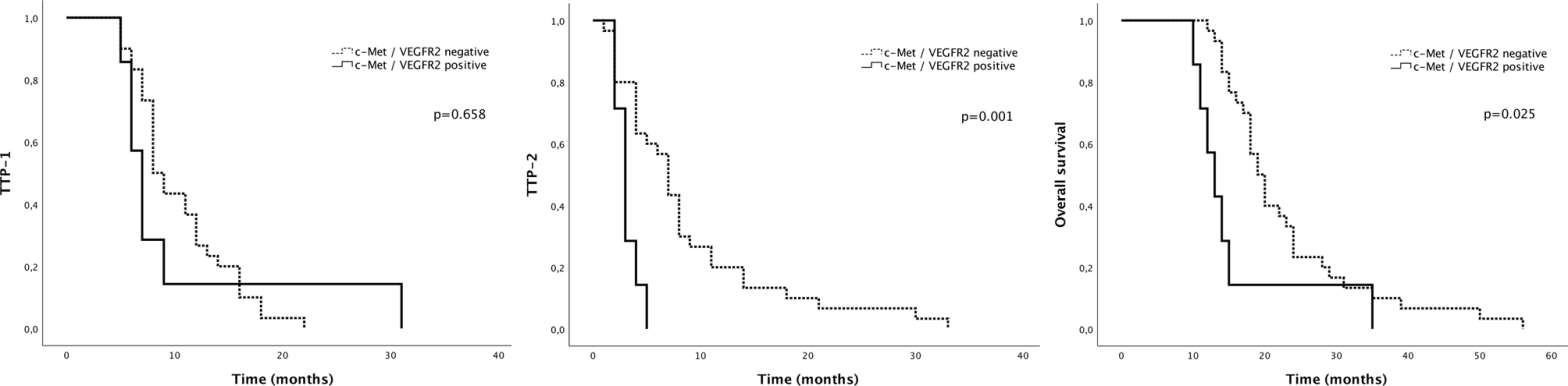
Kaplan–Meier analysis of survival curves according to concomitant c-Met/VEGFR2 expression.

**Figure 4:**
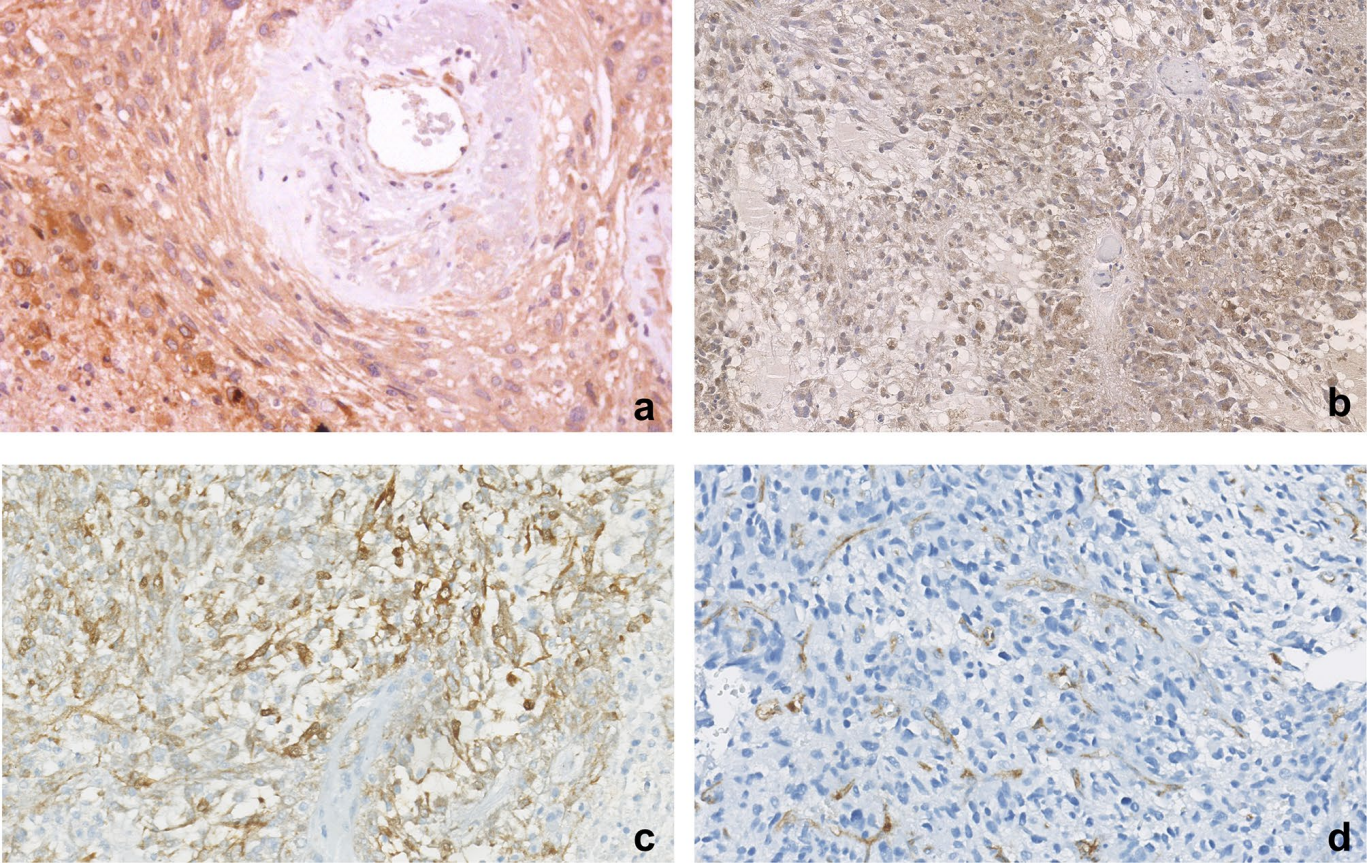
Representative positive immunohistochemical staining with c-Met (**a**), HGF (**b**), VEGFR2 (**c**) and CD31 (**d**) (original magnification × 200).

Concomitant overexpression of c-Met and VEGFR2 was detected in 18.9% (7 of 37) of the patients. This concurrent expression had no impact on TTP-1 (8 versus 7 months, p = 0.658), but was significantly associated with worse TTP-2 (3 months) compared with concomitant negative expression of c-Met and VEGFR2 (7 months, p = 0.001). Concomitant overexpression of c-Met and VEGFR2 was significantly associated with worse OS (13 months) compared with concomitant negative expression of c-Met and VEGFR2 (19 months, p = 0.025) (Fig. 3). Representative images of immunopositivity are depicted in Fig. 4.

A comparative analysis of the clinicopathological features between c-Met positive and negative GBM showed no significant differences concerning age (p = 0.797, Mann–Whitney U test), gender (p = 0.728, Fisher’s exact test), ECOG status (p = 0.167, Mann–Whitney U test), type of surgery (p = 0.240, Chi-square test) or the number of temozolomide cycles (p = 0.148, Mann–Whitney U test). The same was observed when comparing HGF and VEGFR2-positive and negative GBM.

Multivariate analysis of covariates on TTP-2 (including age, ECOG, type of surgery, c-Met and VEGFR2) revealed that c-Met expression (p = 0.045, HR 2.31) and VEGFR2 expression (p = 0.039, HR 2.59) are independent predictors of progression while on bevacizumab. (Table 2) No significant impact on prognosis was observed in OS multivariate analysis.

**Table 2:** Analysis of independent predictors of time to progression of GBM patients while on bevacizumab (TTP-2) based-therapy.

Variable	Multivariate analysis		
	HR	95% CI	p value
**Age**			
≥ 65 versus < 65 years	1.63	0.68–3.93	0.277
**ECOG**			
0–1 versus ≥ 2	0.271	0.07–1.04	0.058
Type of surgery	1.18	0.39–3.50	0.77
c-Met expression	**2.31**	**1.02–5.23**	**0.045**
VEGFR2 expression	**2.59**	**1.05–6.39**	**0.039**

*HR* hazard ratio.

Results indicated in bold are significant

## Discussion

Tumor angiogenesis is essential for tumor growth and invasion, relying on a highly complex network of growth factor signaling, microvascular proliferation, extracellular matrix remodeling, and stromal cell interactions. Despite promising preclinical and clinical results, antiangiogenic therapies led only to mild survival benefits. In GBM, randomized clinical trials (RCT) conducted in the primary setting (AVAglio and RTOG 0825) demonstrated benefits in PFS but no impact on OS. Moreover, the clinical prognostic factors, MGMT status and molecular markers did not seem to be predictive of response^6,7^. On the other hand, the BELOB phase 2 trial supported the use of bevacizumab in combination with lomustine in the recurrent GBM setting, showing a benefit in OS; however, it was not validated in EORTC 26101 phase 3 trial. The latter showed PFS benefit but no OS advantage. Notwithstanding these results, it is worth mentioning that crossovers to off-label use of bevacizumab occurred in 35.5% of the monotherapy group and 18.7% of the combination group^3,8^.

The minimal survival benefits observed in several studies can be secondary to intrinsic or acquired resistance, through the activation of alternative mechanisms that sustain tumor vascularization and growth^15,16^. These mechanisms of resistance can be subdivided into VEGF-dependent alterations, VEGF-independent pathways and stromal cell interactions. Therefore, complementary and novel approaches, such as the combination of these inhibitors with agents targeting alternative mechanisms of blood vessel formation, are urgently needed in order to overcome resistance and improve outcomes^15^.

To date, no subgroup has been identified that consistently benefits from VEGF inhibition^11^. Several putative clinical factors (hypertension and proteinuria)^20^, tissue markers (VEGF-A, VEGFR2, c-Met)^16,21,22^, circulating biomarkers (MMP2)^23^, imaging perfusion and diffusion maps^24^ and molecular markers (EGFR, Proneural subtype)^25–27^ have been investigated, although they lack validation and confirmation among studies.

Some authors have identified c-Met receptor tyrosine kinase as having a role in resistance mechanisms driving GBM invasion in xenografts. This resistance to antiangiogenic agents may be mediated by the upregulation of c-Met gene expression (acquired resistance) or due to a selective survival of subpopulations of tumor cells that overexpress c-Met (intrinsic resistance)^16,28,29^.

In our study we aimed to evaluate the prognostic and therapeutic significance of proangiogenic ligands and receptors (c-Met, HGF, VEGFR2) and MVD, specifically in a clinical population of bevacizumab-treated recurrent GBM patients. Herewith, we attempt to identify which subgroup of patients has an obvious benefit from anti-angiogenic therapy and which subgroup is mainly resistant to this therapy.

Our study detected c-Met overexpression in 36.8% of the patients and revealed a significant reduction in TTP after antiangiogenic therapy, while not affecting TTP after temozolomide. In line with existing evidence that report c-Met overexpression in 34% of GBM^17^, there was a trend towards a shorter OS time in c-Met-overexpressing tumors. This underscores the role of c-Met in early resistance and tumor invasiveness. VEGFR2 overexpression by tumor cells was detected in 25.6% of the patients and – similarly to c-Met—was associated only with a shorter TTP after antiangiogenic therapy. Kessler et al. detected VEGFR2 immunopositivity in 19% of the specimens and showed increased aggressiveness and shorter survival associated with VEGFR2 overexpression in glioma cells, by hampering antiangiogenesis and inducing a proinvasive response to bevacizumab^21^.

Although some authors^30^ have found in vitro and in vivo evidence that HGF co-expression is a key predictor of sensitivity to c-Met inhibitors, in our study HGF expression was not predictive of differences in TTP-1, TTP-2 and OS.

Tumor MVD is reported as a potential predictor of bevacizumab benefit in other malignancies, such as ovarian cancer, showing a positive association between MVD and magnitude of bevacizumab effect^31^. In GBM, Tamura et al. showed that antiangiogenic therapy induces the apparent vascular normalization and that peritumoral immature vessels may be resistant to treatment with VEGFR-targeting drugs^32^. In our study we did not find any association between MVD and TTP or OS. Other markers, such as endoglin, are highly expressed on actively proliferating endothelial cells and may increase specificity in angiogenesis evaluation in GBM^33^.

Despite some studies reporting significant associations and promising predictive markers, others have not accomplished the same conclusions. Kopp et al. studied proliferation, molecular, vascular, and radiological markers in 33 patients with recurrent GBM treated with bevacizumab in the first recurrence, but did not find any predictive biomarker of response or survival benefit for antiangiogenic therapy^34^.

Remarkably, the worst TTP-2 and OS was found in GBM with concomitant overexpression of c-Met and VEGFR2, which denotes primary/innate activation of both angiogenesis pathways. This data emphasizes the rational of combining angiogenesis inhibition with drugs that target evasive resistance mechanisms to provide more durable efficacy. In this perspective, c-Met upregulation seems to be a putative major factor for antiangiogenic resistance. Therapeutically targeting c-Met and disrupting c-Met/HGF pathway alone or in combination with antiangiogenics may be a strategy to prevent or overcome resistance and enhance therapeutic efficacy.

In vitro and in vivo synergistic effect of anti-MET and VEGF combination therapy for MET-overexpressing GBM has been reported^35^. At this time, several drugs targeting c-Met are available in clinical trials^36^. Selecting the best combination therapy and subgroup of patients may be the key to overcome resistance and invasiveness in GBM. Some recent clinical trials involving onartuzumab^37^ and capmatinib^38^ uncovered no clinical benefit, but demand more stringent molecular selection strategies. RCT analyzes highlight significant heterogeneity regarding co-occurring genetic alterations that are consistently described in glioblastoma landscape. Additionally, both MET copy number and protein overexpression may have predictive value, but appropriate cut-offs need to be established. Also, the extent of brain exposure to MET inhibition is unknown and may affect outcome. Others have suggested that HGF expression or MGMT unmethylated status may confer benefit. Other RCT are currently ongoing, such as NCT02386826, which addresses the combination of INC280 (capmatinib) and bevacizumab.

Our study adds valuable data to the current body of evidence, suggesting that patient stratification according to the expression of angiogenesis-related receptors may predict antiangiogenic treatment efficacy. Indeed, in this series of patients, c-Met and VEGFR2 were noticeably associated with differences in survival under bevacizumab therapy, while HGF and MVD did not display any impact.

As limitations of our study we should point out that it is a retrospective study with a limited number of patients, which may have precluded some significant associations. Also, MGMT methylation was not consistently available. Nonetheless, a thorough pathological and IHC evaluation, detailed clinical, therapeutic, and survival data uncovered significant data.

## Conclusion

Our data substantiate the assumption that c-Met and VEGFR2 overexpression might play a role in developing GBM early resistance and are associated with worse response to antiangiogenic therapies. There is a need for additional studies with prospective validation of these putative biomarkers in the diagnostic stratification of recurrent GBM clinical trials.

## Data Availability

The datasets generated and/or analyzed during the current study are not publicly available but are available from the corresponding author upon request.
